# To signal or not to signal? Chemical communication by urine-borne signals mirrors sexual conflict in crayfish

**DOI:** 10.1186/1741-7007-8-25

**Published:** 2010-03-30

**Authors:** Fiona C Berry, Thomas Breithaupt

**Affiliations:** 1 University of Hull, Department of Biological Sciences, Hull, UK

## Abstract

**Background:**

Sexual selection theory predicts that females, being the limiting sex, invest less in courtship signals than males. However, when chemical signals are involved it is often the female that initiates mating by producing stimuli that inform about sex and/or receptivity. This apparent contradiction has been discussed in the literature as 'the female pheromone fallacy'. Because the release of chemical stimuli may not have evolved to elicit the male's courtship response, whether these female stimuli represent signals remains an open question. Using techniques to visualise and block release of urine, we studied the role of urine signals during fighting and mating interactions of crayfish (*Pacifastacus leniusculus*). Test individuals were blindfolded to exclude visual disturbance from dye release and artificial urine introduction.

**Results:**

Staged female-male pairings during the reproductive season often resulted in male mating attempts. Blocking female urine release in such pairings prevented any male courtship behaviour. Artificial introduction of female urine re-established male mating attempts. Urine visualisation showed that female urine release coincides with aggressive behaviours but not with female submissive behaviour in reproductive interactions as well as in intersexual and intrasexual fights. In reproductive interactions, females predominately released urine during precopulatory aggression; males subsequently released significantly less urine during mating than in fights.

**Conclusions:**

Urine-blocking experiments demonstrate that female urine contains sex-specific components that elicit male mating behaviour. The coincidence of chemical signalling and aggressive behaviour in both females and males suggests that urine release has evolved as an aggressive signal in both sexes of crayfish. By limiting urine release to aggressive behaviours in reproductive interactions females challenge their potential mating partners at the same time as they trigger a sexual response. These double messages should favour stronger males that are able to overcome the resistance of the female. We conclude that the difference between the sexes in disclosing urine-borne information reflects their conflicting interests in reproduction. Males discontinue aggressive urine signalling in order to increase their chances of mating. Females resume urine signalling in connection with aggressive behaviour, potentially repelling low quality or sexually inactive males while favouring reproduction with high quality males.

## Background

Sexual selection theory predicts that females will minimise risk and energy expenditure during courtship, due to their higher investment in offspring compared to males [1]. Asymmetry in the evolutionary interests of males and females can result in sexual conflicts over whether or not mating takes place [2]. Males try to maximise the number of mating opportunities while females are highly selective in choosing when and with whom to mate [3]. Consequently males generally perform the more costly role in pair formation and invest in courtship signals to compete for mating opportunities with the choosier sex. Previous research has primarily focused on the role of sexual selection in shaping acoustic and visual advertisement signals with limited effort directed at the role of chemical signalling in courtship behaviour [1].

Chemical communication systems, such as those of moths [4] and decapod crustaceans [5], often involve a female signaller and male receiver [6,7]. Females appear to initiate courtship through the release of a sex pheromone that triggers the male's mate search and/or courtship behaviours. Modern definitions of communication emphasise that a stimulus is a signal only if it: (i) affects the behaviour of other organisms, (ii) evolved because of those effects, and (iii) is effective because the response has evolved to be affected by it [8,9]. Williams [10] questioned the existence of female pheromone signals, such as those produced by moths, based on the fact that they are 'produced in minute traces' and 'not by machinery designed by selection to produce a male response'. His view, particularly with respect to insect chemical communication, has been opposed by arguments emphasising the adaptive value of female signalling [11]: by releasing minimal amounts of pheromone, female moths impose scramble competition between males resulting in the selection of males with good searching and chemosensory abilities [12]. Furthermore, female moths releasing pheromones bear a lower predation risk than mate-searching males since a specific pheromone stimulus may be less conspicuous to their main predators (birds, bats) than the visual/acoustic stimulus of a moving male [13].

This reasoning does not explain female pheromone signalling in aquatic environments. Unlike aerial predators, many aquatic predators use chemical stimuli to detect their prey, imposing a high risk to a chemical signaller in water [14,15]. In decapod crustaceans there is no evidence for improved chemosensory abilities of males as a result of scramble competition. Yet, evidence from several species of decapod crustaceans suggests that females release urinary signals eliciting specific male responses [5]. Responses include near-field attraction [16,17] and courtship behaviour [18]. In crustaceans, evidence in support of a communication route from female to male is predominantly based on the analyses of male responses; little is known about female signal production in relation to courtship behaviours (see [19]).

Here, we investigate in the signal crayfish (*Pacifastacus leniusculus*) the function of urine signalling in reproductive interactions by studying the context of release as well as the receiver response. Crayfish live at high population densities and have multiple opportunities to mate [20]. Females invest considerable energy in rearing their offspring [21]. Female signal crayfish spawn in the autumn and then provide sole parental care for up to 6 months. Male crayfish, in contrast, despite their initial investment in producing spermatophores are able to inseminate multiple females and are not involved in brood care [20]. The great imbalance between the sexes in investment into offspring suggests that, in crayfish, sexual conflict over mating may be particularly strong. Hence, it is not surprising that mating in crayfish is generally preceded by fights, with females trying to resist male mating attempts [20,22,23]. In this scenario, it is expected that females do not invest in courtship signals as they may encourage rather than discourage male mating attempts. However, a recent study demonstrated that both male and female rusty crayfish (*Orconectes rusticus*) release urine during reproductive interactions [19]. Furthermore, experimental evidence suggests that female odour may elicit male courtship behaviour [24,25]. Odour from receptive females was more effective in changing male behaviour than chemical stimuli from males or juvenile females. In these studies the female stimuli were released from inanimate sources and did not elicit male mating attempts comparable to natural courtship behaviours.

Our study addresses the obvious discrepancy between the experimental evidence suggesting female chemical courtship signals and the reported resistance of female crayfish to mating, which is in line with sexual conflict theory. We study the function of female urine signals by investigating both the effect on the male receiver as well as the behavioural context of female signalling. Male responses are assessed in staged male-female interactions by blocking and artificially introducing the urine of receptive females. By visualising the urinary release in male and female crayfish (*P. leniusculus*) we investigate whether urine signalling in reproductive interactions differs from signalling in aggressive interactions. Our data show that female urine, while instrumental in initiating male courtship, is released in an aggressive context. The results suggest that in reproductive interactions, driven by conflicting interests between the sexes, the function of urine signals can be different between males and females.

## Results

### Effect of female urine on male mating attempts

In 15 blindfolded but otherwise unrestrained control pairs, 7 males showed mating attempts by seizing (7 males), turning (6 males), and mounting (1 male) the female. When female nephropores were blocked and water was introduced, none of the males showed any courtship behaviour. All interactions were of an aggressive nature. Artificial introduction of female urine in interactions with urine-blocked females stimulated male mating attempts in six encounters. Six males seized the females, which is a significant increase in mating attempts compared to urine-blocked pairs stimulated with water (*P *= 0.017; Fisher exact test). Five males turned the female so ventral surfaces were facing, which is a significant increase compared to no cases of turning in urine-blocked pairs (*P *= 0.04; Fisher exact test). Mounting was observed in two experiments.

### Sex-specific differences in chemical signalling

**Figure 1:**
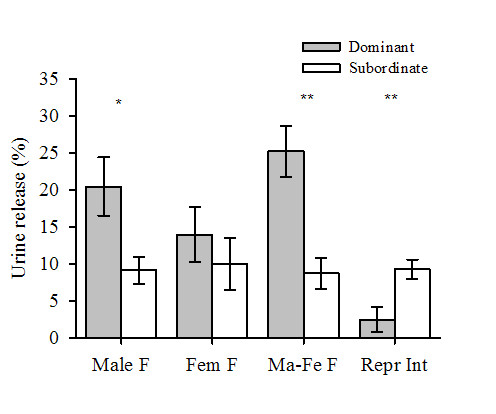
**Mean (± standard error of the mean (SEM)) urine release by dominant (grey bars) and subordinate (white bars) animals in male fights (Male F), female fights (Fem F), mixed-sex fights (Ma-Fe F) and reproductive interactions (Repr Int)**. Male crayfish were labelled as dominant animals (grey bars) in reproductive interactions. Asterisks indicate differences between interactants (*P < 0.05, **P < 0.01, paired t test).

**Figure 2:**
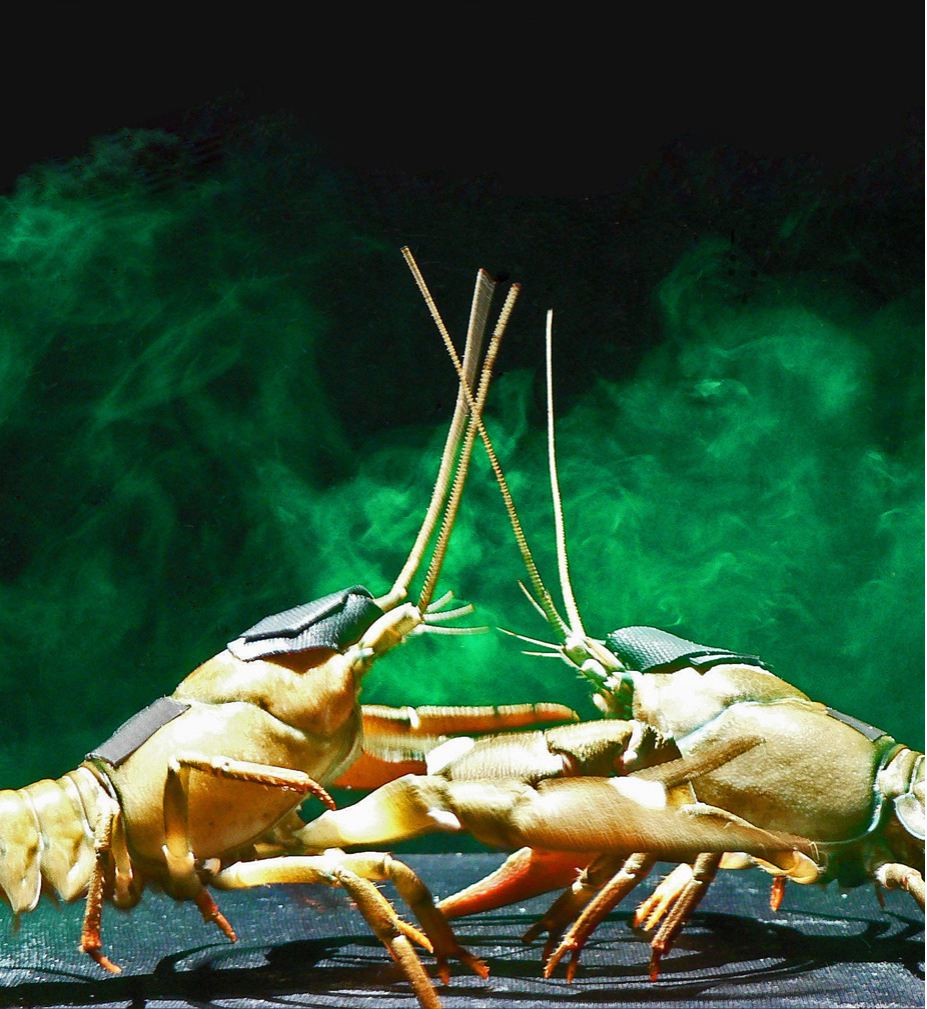
**Visualisation of urine release in a fight between two male signal crayfish**. Both animals had been injected with fluorescein dye, which accumulates in the bladder staining the urine. Urine is released from the nephropores and dispersed by the frontally projecting gill currents. The highest urine concentration is near the antennular chemoreceptors. The antennules are the small forward pointing antennae. Three of the four bilateral antennular flagella are visible in each animal. Crayfish are reversibly blindfolded by black pond liner wrapped around the rostrum and eyestalks and fixed to the dorsal carapace. Black tape on the posterior carapace was used to seal the hole left by fluorescein injection.

Crayfish displayed sex-specific differences in urine release in agonistic and reproductive interactions (F_2,117 _= 8.8, *P *< 0.001, two-way analysis of variance (ANOVA); Table 1, Figure 1). Males engaged in reproductive interactions released urine for a significantly shorter time in comparison to dominant males when fighting, or to females that were fighting or involved in reproductive interactions (*P *< 0.05, Tukey honestly significant difference (HSD) test, Table 1; Figure 2). Females, in contrast, released urine for a similar duration irrespective of their dominance status in a fight or whether they were engaged in reproductive interactions (Table 1).

**Table 1:** Influence of sex and behavioural state on urine release

Level	Least squares mean	Standard error	Tukey HSD test
Male dominant	28.0	1.9	A
Female dominant	18.4	2.6	B
Female mating	16.5	2.6	B
Male subordinate	16.0	2.6	BC
Female subordinate	15.0	1.9	B
Male mating	5.5	2.6	C

Results from *post hoc *analysis of two-way analysis of variance (ANOVA) analysing interaction effects of behavioural state (dominant, subordinate, reproductive interaction) and sex on duration of urine release. Least square means and standard error are adjusted values for average urine release time as predicted by the general linear model when all other factors are controlled for. Levels not connected by the same letter are significantly different (*P *< 0.05, *post hoc *Tukey honestly significant difference (HSD) test).

### Mating behaviour

Reproductive interactions of fluorescein-injected pairs were analysed in detail and followed a characteristic pattern that has been previously observed in *P. leniusculus *[25] taking on average 23 min to complete.

**Figure 3:**
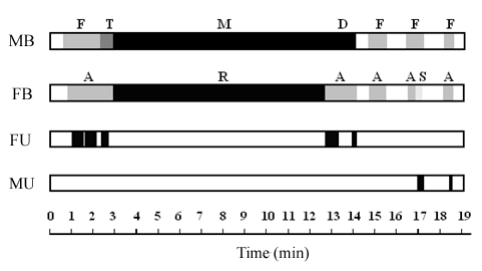
**Ethogram of a sexual interaction between signal crayfish**. Male behaviours (MB) displayed were fighting (F) (behavioural levels 1 to 5, see Table 2); seizing/turning (T); mounting/deposition (M) and dismounting (D). Female behaviours (FB) shown were aggressive (A); receptive (R) and submissive (S); see Methods for description of behaviours. White sections on the top two strips show times when animals were separate. Female and male urine release (FU, MU) is denoted by black bars on the lower strips. Urine release was associated with aggressive behaviours from female crayfish.

During reproductive interactions agonistic behaviours generally preceded mating and could reach levels of unrestrained aggression (Level 5; Table 2). Mating commenced when the male seized the female at the rostrum, antennae or chelae and tried to turn her so that her ventral surface was facing him and spermatophores could be deposited. Females exhibited aggressive behaviours prior to and at the onset of mating but showed receptive postures before the males could successfully deposit spermatophores (Figure 3).

**Table 2:** Definition of agonistic and sexual behaviours

Behaviours	Agonistic level	Behavioural elements
**Agonistic behaviours:**		
Fleeing	-2	Tail flipping, walking away quickly
Avoidance	-1	Walking away slowly, turning away from opponent
Separate	0	Animals separate
Initiation	1	Approach or following opponent, turn towards opponent
Threat display	2	High on legs, meral spreading
Touching	3a	Animals touching via body, antenna or chela(e) with limited movement
Physical contact (claws do not grasp)	3b	Antenna whipping, claw pushing, claw boxing, claw tapping
Physical contact (claws grasp)	4	Claw lock, clamping chela(e) onto opponents body
Unrestrained aggression	5	Claw snapping, claw ripping
		
**Mating behaviours:**		
Seizing	N/A	Male grips the female at the rostrum, chela(e) and/or antenna, usually from an angled position
Turning	N/A	Male secures pereopods around the cephalothorax of the female (either from an adjacent position or by climbing on top of the female) and turns her ventral side up
Mounting	N/A	Male holds female so ventral surfaces are facing and maintained in a parallel position
Spermatophore deposition	N/A	Arching and depression of the male abdomen while depositing spermatophores on female ventral surface. Pauses common between cycles.
Dismount	N/A	Female is released from the mounting position through movement of the pereopods or chela(e)

For full descriptions of behaviours, see [25,44].

### Female urine release in reproductive interactions

**Figure 4:**
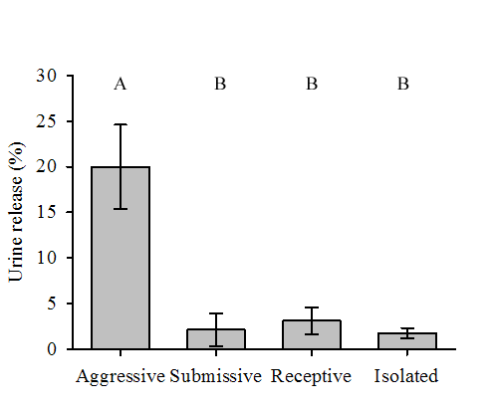
**Urine release by female crayfish during different categories of behaviours**. Values are mean urine release as a percentage of total time spent displaying each behaviour (± standard error of the mean (SEM)). Behaviours not labelled by the same letter denote significant differences (*P *< 0.05, *post hoc *Tukey test).

All females released urine prior to mating, with 94% of females releasing urine during precopulatory aggression. In comparison a third of males did not release any urine prior to mating. Urine release by females primarily occurred during aggressive behaviours in relation to other behaviours (Figure 4; χ^2 ^= 27.14, degrees of freedom (df) = 3, *P *< 0.001; Friedman test). Females released urine for longer while displaying aggressive behaviours in comparison to receptive or submissive behaviours (*P *< 0.05; Tukey test), or periods where they were not engaged in social interaction (*P *< 0.05; Tukey test).

### Agonistic behaviour

Fights between female crayfish and mixed-sex fights both followed the characteristic pattern that has been previously described for adult male crayfish [26,27]. The winner of a fight displayed highly aggressive behaviours (Table 2; levels 3b to 5) in comparison to the subordinate animal that displayed submissive behaviours (levels -1, -2) and did not re-engage in fights once dominance had been established.

There was a significant difference in the proportion of time crayfish spent displaying highly aggressive behaviours (high aggression index) between different fights (*F*_2,87 _= 0.67, *P *< 0.001; one-way ANOVA). Male fights were more aggressive than female fights (*P *< 0.05; Tukey HSD test) and mixed-sex fights (*P *< 0.05; Tukey HSD test), whereas similar levels of aggression were shown in female fights and mixed-sex fights. Females were always subordinate in mixed-sex fights, probably due to their smaller claw size.

In fight interactions urine release was associated with aggressive behaviours in intrasexual fights but not in intersexual fights, with urine released for longer durations by individuals displaying a high aggression index (Pearson correlation: male fights, *P *= 0.038, *r *= 0.39, N = 29; female fights, *P *= 0.001, *r *= 0.59, N = 30).

## Discussion

Previous studies have suggested that urinary cues play a key role in coordinating mating behaviour of decapod crustaceans [5]. In most cases it is believed that female urine triggers the start of courtship behaviour. This contradicts expectation from sexual selection theory, as females are the limiting sex and should invest little in courtship. Here we confirm that in *P. leniusculus*, a crayfish species with a strong disproportion in reproductive investment between the sexes, the females triggers courtship behaviour by sending urinary chemical signals. Males attempt to mate only if they receive urinary signals from the female. The context of female signalling, in contrast, is aggressive. Female urine release coincides with aggressive rather than reproductive or submissive behaviours (Figure 4). Both males and females release urinary signals in same-sex and mixed-sex fights (Figure 1). Males reduce or discontinue urine release as they switch from aggressive to courtship behaviours (Table 1, Figures 1 and 3). Our results suggest that the release of urine in social interactions by crayfish has evolved as an aggressive signal in both males and females rather than a courtship signal.

### The role of female urinary signals in eliciting male courtship

Blocking female urine abolished any male mating attempts. Mating behaviours were displayed only when female urine was artificially released or when female nephropores were not blocked. When urine was introduced the likelihood of courtship or mating (6 out of 15 pairs showed mating attempts) was similar to mating or courtship success generally found when staging intersexual encounters for a limited time in a small aquarium (7 out of 15 in control experiments; in other experiments 7 matings were recorded in 17 pairings; Fiona C. Berry, unpublished results). Hence, female urine release appears to be essential in stimulating male mating attempts. Urine visualisation experiments demonstrate that notwithstanding the aggressive context of female signal release, male courtship behaviour (seizing, turning) was always preceded by female urine delivery. After females released urine, males not only started courtship activity but also reduced their own level of aggressive urine signalling. This suggests that female urine is instrumental in diverting male behaviour from aggression to courtship. This conclusion is consistent with previous studies of crayfish (*P. leniusculus*), showing that stimulation with exogenous urine (or conditioned water) from receptive females is more potent in eliciting behavioural and physiological responses in males than urinary chemical stimuli from males or juvenile females [24,25,28].

The female's physical aggression towards males, coupled with a urine stimulus indicating receptivity, sends contradictory messages to the male. How could this ambivalent behaviour evolve? Is it adaptive to the female? Females could profit in different ways from displaying such conflicting signals. Crayfish live at high population densities and during the breeding season females have multiple encounters with adult conspecifics. Some of these encounters may be of agonistic nature (for example, in the presence of a food), others may be sexually motivated. The relative low rate of mating in our experiments indicates that even sexually mature males do not always respond to female urinary signals with courtship behaviours. Therefore, the aggressive behaviour covering up the release of sexually attractive chemicals will be adaptive in many types of interactions that involve competition over resources. Moreover, females may execute mate selection by sending these conflicting signals as discussed further below.

### Male urine signalling and female mate choice

Females are expected to prefer to mate with dominant rather than with subordinate males. In crayfish fights, dominant males release more urine than the subordinate (Figure 1; [26]). Hence, it is expected that during courtship male crayfish actively advertise their dominance to the female through chemical signals, in order to maximise their chance of mating. Cockroaches, for example, advertise their dominance status to females using the same chemical components in courtship as in aggressive interactions [29,30]. However, the results of our study contradict this in crayfish because males significantly reduced their urine release when interacting with a female during the breeding season (Figure 1, Table 1). In all, 30% of the crayfish males did not release any urine at all during reproductive interactions suggesting that females do not use urinary chemical cues to assess male quality. A recent study [31] showed that female red swamp crayfish (*Procambarus clarkii*) do not show a preference when allowed to choose between an unfamiliar dominant versus unfamiliar subordinate male of similar size. Similarly, female signal crayfish show no preference for a dominant male if given a free choice between males (Fiona C. Berry, Adam D. Smith and Thomas Breithaupt, unpublished results). However, when allowed to watch the males in agonistic interactions prior to the choice, female *P. clarkii *subsequently showed a preference for the dominant male. We conclude from these studies, that males do not actively advertise their quality (dominance status or size) by urinary chemical signals.

However, recent evidence suggests that female *P. clarkii *use combined visual and chemical information to identify the sex of conspecifics. Females were shown to more readily approach larger males and to discriminate between individuals of opposite sex only if they received bimodal (chemical and visual) information [32,33]. During the nocturnal activity period of crayfish visual information is strongly restricted. While chemical information alone is not sufficient for the female to identify sex or assess size [32] tactile sensory input during physical interactions may provide crucial information to the female for assessment of male quality. Therefore, by engaging in aggressive physical interactions, females can assess male size and strength using mechanoreceptors.

The reduction of male urine release during courtship is surprising but could be regarded as an appeasement towards the female. It may serve the male to de-escalate female aggression and to increase female motivation to mate.

### Sexual conflict and urine signalling

For most animal species there is unequal investment in gametes and progeny between the sexes, with males investing less than females [3]. Males consequently have a higher potential reproductive rate and compete over females, while females are more resistant and are interested only in mating with the best available male [34]. Strong asymmetries between the evolutionary interests of the sexes inevitably results in sexual conflicts [35].

Sexual conflict is expected to be particularly strong in crayfish due to the considerable difference between the sexes with regard to their investment into offspring [20]. In such mating systems, the male generally does most of the signalling or advertisement while the female chooses among the signallers [36]. Receptive females are not expected to actively advertise their receptivity with urine signals. Instead, the display of precopulatory aggression indicates resistance of the female to male mating attempts and female urine release occurs as an integral component of aggressive behaviour [26].

Urine release during fighting behaviour follows the same pattern in males and females, suggesting a function as aggressive signal in both sexes. Urine release, therefore, does not appear to be under 'sexually antagonistic coevolution' [2].

Males are under high selection pressure to detect sexually receptive females and to break their resistance to mate. Equipped with larger claws relative to females of similar body size, males are able to coerce some females to mate [37], giving them the active role in reproduction. At the high population densities typical for crayfish, males regularly encounter females. Detection of sexually receptive females at the start of an agonistic interaction is crucial in order to utilise the mating opportunity. Once the female is detected, males need to overcome their resistance. By discontinuing their own aggressive urine signalling males may mitigate the female's aggressive behaviour and facilitate mating.

Females, in contrast, are expected to be more selective, mate less frequently and only with high quality males. By linking urine release with aggressive behaviours females generate conditions that allow them to conduct mate choice. In encounters with sexually active males, urine metabolites will initiate male mating attempts while the aggressive behaviour will test and challenge the male. By means of the physical interaction females can assess the size and strength of the male. After mating, females exhibit mate choice, either by physically removing unwanted spermatophores (Fiona C. Berry, unpublished results) or by adjusting their reproductive effort in relation to male traits. Galeotti and coworkers showed, that female crayfish *Austropotamobius italicus *produce larger but fewer eggs for small males with large claws and more numerous but smaller eggs for large-sized, small-clawed males [37]. Similarly, female *P. clarkii *invest in larger eggs after having copulated with large males [38]. In addition, the resistance of the female may itself be conducive to mate selection. Larger and stronger (high quality) males may be more successful in overcoming the resistance of the females than small or weak males. Hence, by linking urine release with aggressive behaviour females can select high quality mates and enhance their fitness.

### The 'dilemma' of urine signalling

As a source of pheromone communication urine can convey multiple messages, with likely uncheatable information about the physiological state and identity of the sender. Our study shows that the urine of female crayfish during the breeding season gives away information about her receptivity, which can be exploited by conspecific males. This signalling strategy can be disadvantageous for the female when engaging in interactions with low quality males. Perception of sexual metabolites in the urine may increase the persistence of males in interacting with the female, entailing additional time and energy costs for the female, which are not balanced by reproductive benefits. Many animals including a range of mammals and fish release urine as a means of communication [39,40]. Releasing urine always carries the risk of revealing unwanted information about the state of the signaller and hence is prone to exploitation. Crustaceans can adjust the timing of urine release by the opening and closing of the sphincter muscle of the nephropore. However, they may not be able to control the chemical composition as the bladder accumulates urine over extended time periods (from minutes to hours). Receivers are expected to use information provided by the metabolites in the urine as this may give them a competitive or reproductive advantage. Hormonal metabolites carrying information about sexual receptivity were suggested to be particularly prone to detection by 'spying' conspecifics [41,42].

Female crayfish, on average, do not gain any non-reproductive benefit by courting or mating. Therefore, the urine signalling system may be designed to provide conditions facilitating mate choice. In contrast to crayfish, in many other crustaceans (for example, American lobsters, shore crabs) mating is linked to female moulting [35]. Moulting is the most vulnerable time period in a crustacean's life. Moulted crustaceans are potential prey to conspecifics as well as predators and females attract males and are guarded throughout this vulnerable time. Recent studies have shown that a urinary component released by female shore crab is a feeding deterrent to males but not to conspecific females [43]. In such cases, females may gain an advantage by attracting males through urinary signals. Other examples of urine-borne female sex pheromones including crustaceans, fish and mammals need to be explored to investigate how signal exploitation is mitigated by adjusting the timing of urine release.

## Conclusions

A still unresolved question in animal communication research is why chemical signals that initiate courtship (sex pheromones) are normally emitted by females and not by males. In courtship interactions mediated by other communication channels (visual, acoustic, electric) it is generally the male producing the advertisement signal and the female, as the limiting sex, displaying a choice in responding or not. Our study provides evidence, that in crayfish (*P. leniusculus)*, urine-borne chemical signals of the female initiate male courtship. Urine release, in both males and females, is always linked to aggressive behaviour suggesting that urine signalling has evolved as an aggressive signal in both sexes. In reproductive interactions, females show resistance to the males and release urine during the fight preceding mating. Males, after receiving female urine pulses, switch from aggression to courtship behaviour and significantly reduce their urine output. These findings suggest that crayfish carefully adjust the timing of urine release in order to reduce the potentially costly consequences of providing multiple sources of information to a receiver with contrary interest. Males, by discontinuing their own aggressive urine signalling may mitigate the female's aggressive behaviour and enhance mating success. Females, by linking urine release to aggressive behaviours provide conditions that enable them to assess the size and strengths of the male, facilitating postcopulatory mate choice.

## Methods

### Animals

Adult crayfish (*P. leniusculus*) were obtained in September 2005, March 2006, August 2007, and August 2008 from a crayfish dealer (Chris Campbell, Milton-on-Stour, UK). Animals were separated by sex and a maximum of 25 animals were kept in a communal holding tank (91.5 × 30 × 30 cm), containing carbon-filtered water. Males were physically but not chemically separated from females. Crayfish were maintained at 10°C, 10:14 h light/dark cycle (breeding season; September to December) and 14°C, 12:12 h light/dark (March, August). Individuals were never used in more than one experimental trial.

Within the breeding season, prior to social interaction trials individuals were tested for sexual receptivity. Male crayfish were classed as sexually receptive if they tried to turn or mount the female while female crayfish were identified as sexually receptive if glair glands were visible as whitened tissue on the underside of the telson. Unreceptive crayfish were not used in sexual interaction trials.

### Urine blocking experiments

Experiments were conducted in November during the breeding season using sexually receptive male and female crayfish. At 1 week prior to the experiments animals were isolated in individual plastic containers (24 × 18 × 8 cm) and stored in a temperature controlled room at 11°C, mimicking the seasonal temperature in the natural environment. Females used were slightly smaller (mean ± standard error (SE) carapace size 35.3 ± 0.5 mm, N = 30) than males (37.1 ± 0.5 mm, N = 30). Animals were blindfolded 24 h prior to the experiment by wrapping opaque plastic (1 × 4 cm) around the eyestalks and rostrum and securing excess material to the carapace using cyanoacrylate glue. Blindfolding served to prevent disturbance of animals during the experiments by the observer introducing stimuli through a syringe.

#### Experimental design

A urine-blocked female was paired with an unblocked male for 1 h. Female urine (urine treatment, 15 pairs) or filtered tap water (water treatment, 15 pairs) was introduced by a 250 μl Hamilton syringe between the 2 animals in 5 to 8 pulses of 25 to 50 μl separated by at least 10 s when animals were facing each other and aggressively interacting. In a control experiment (N = 15), an unblocked male and an unblocked female were paired for 1 h without introducing other stimuli. Treatment and control experiments were alternated to ensure that all experimental conditions were tested throughout the reproductive season.

#### Urine blocking

At 3 h before experimental treatment, female nephropores were blocked by attaching 1 cm of silicon tubing (1.6 mm diameter, Bio-Rad Laboratories, Hemel Hempstead, UK) to the basal segment of each second antenna covering the nephropore, using cyanoacrylate glue (Zap-a-Gap, Pacer Technologies, Rancho Cucamonga, USA). To ensure the tubing was secure an additional cyanoacrylate layer was applied around the tube and dried using an accelerator fluid (Zip kicker, Pacer). The open end of the tubing was sealed with plasticine and the seal was reinforced by cyanocrylate glue and accelerator. Dye studies were conducted to ensure that the plasticine plug was efficient at blocking urine release from interacting crayfish.

#### Stimulus urine and water

Urine was collected from the nephropores of receptive females that were not used for the experiments. Females were strapped to a board using elastic bands tied around the board and the crayfish tail and claws. Any water was cleared from the nephropores and the surrounding area so it could not contaminate the urine sample. The urine was then extracted using a vacuum pump connected to Teflon tubing (1.5 mm diameter) and a 1.5 ml collection vial. At least 200 μl was collected from each of 15 different females and kept in a freezer at -67°C. Prior to the experiments, samples were thawed and stored at room temperature for 1 h directly before to the experiment. Stimulus water used in the experiment was filtered through a 25 cm presediment filter followed by a 25 cm activated carbon filter (Pozzani Pure Water, Louth, UK) and stored at room temperature 1 h prior to the experiment.

#### Experimental procedure

Individuals were introduced to both sides of a central acrylic divider in an aquarium (30 × 20 × 20 cm). After 10 min acclimation the divider was lifted and animals were allowed to interact for 1 h. Following each experiment, the tanks and dividers were washed thoroughly using carbon-filtered water. Interactions were recorded from the side with a Panasonic camcorder (NV-GS180EB, Panasonic U.K. Ltd., Bracknell, UK) and a Sony DVD recorder (VRD-MC5, Sony U.K. Ltd., Basingstoke, UK) for later analysis. Video recordings were analysed by an independent observer blind to the experimental treatment. The presence of unambiguous male courtship behaviours was noted in each experiment including seizing, turning and mating (mounting with or without spermatophore deposition, see Table 2 for definitions of behaviours).

### Urine visualisation experiments

To study social interactions we used 60 intermoult female crayfish (mean ± SE carapace size of 34.7 ± 0.2 mm, mass 29.1 ± 0.6 g) and 60 intermoult male crayfish (mean ± SE carapace size 36.3 ± 0.3 mm, mass 33.8 ± 0.9 g), with intact appendages. Male fights, female fights and reproductive behaviours were studied within the breeding season (October to December) whereas male-female fights were observed out of the breeding season (March, August). To eliminate the effects of body size on social interactions crayfish were size matched. In same-sex fights carapace and chelae length differences were less than 5%, while for mixed-sex experiments animals were matched only for carapace length due to male-females chelae asymmetries (within 5% for mixed-sex fights and 10% for reproductive interactions). At 1 week prior to interactions individual crayfish were isolated in separate 3 l plastic containers (24 × 17.5 × 8 cm). Animals were blindfolded 24 h prior to the experiment by wrapping opaque plastic (1 × 4 cm) around the eyestalks and rostrum and securing excess material to the carapace using cyanoacrylate glue.

#### Urine visualisation procedure

Fluorescein was used to visualise urine release following the methods developed by Breithaupt and Eger [26]. A 0.3% sodium fluorescein solution (dose 9 to 10 μg/g body mass) was injected into the pericardium region of crayfish 3 to 4 h prior to experiments using a 250 μl syringe (Hamilton Bonaduz AG, Bonaduz, Switzerland) and a 45-gauge needle (BD Microlance™, Drogheda, Ireland). After injection the hole was sealed using plasticine and tape to avoid haemolymph loss, and crayfish were fed on defrosted prawns. The technique was successful in visualising urine in all individuals (N = 120; see Figure 2 for an example of urine visualisation in two males; see also Additional file 1 for original data used to perform the analysis).

#### Interaction procedure

Interactions took place in a glass aquarium (40 × 20 × 20 cm) adapted for filming fluorescein release by covering the walls with black opaque lining. Light from a 250 W slide projector was reflected from the top into the tank by a mirror (44 × 20 cm). Interactions were filmed with a camcorder (Sony Hi8, CCD-VX1E or Panasonic NV-GS180EB) from a front view only. Interactions started after a 30 min acclimation period, where animals were physically and chemically isolated by an opaque divider. Fights were recorded for 30 min after the divider was lifted; reproductive interactions were recorded until 5 min after mating ended (defined as when an animal dismounted and mating behaviour did not reoccur after 5 min). Following each experiment the tanks and dividers were washed thoroughly using carbon-filtered water.

#### Urine release analysis

Recorded interactions were analysed using a behavioural software package (The Observer V. 5.0, Noldus, Leesburg, VA, USA). The timing of urine release and the behaviour of each crayfish was analysed in 15 male-male, 15 female-female and 15 male-female fights and 15 male-female reproductive interactions. The release of stained urine was recorded for both individuals during the acclimation and experimental period. A measure of urine output was determined for each individual from the time spent releasing urine (% of time spent releasing urine in relation to the total time animals were in contact).

#### Behavioural analysis

Throughout the experimental phase both crayfish were assigned a behavioural score using a mutually exclusive scale, incorporating agonistic (adapted from [26]) and reproductive behaviours (adapted from [25]) (Table 2). For each interaction dominant animals were identified as those that initiated fights and showed high levels of aggression (grasping of opponents body with the claws, unrestrained aggression). Subordinate animals displayed submissive behaviours (avoidance, fleeing) and did not reengage in fights.

Female crayfish were scored during reproductive interactions to assess their motivation towards mating. Females were scored as: (1) receptive, where females stretch their claws out in front of their body, which is lowered towards the substrate, aiding the male in mounting and spermatophore deposition; (2) submissive, where the female flees or avoids the male (levels -2, -1. Table 2); (3) aggressive, where the female displays characteristic aggressive behaviours found in fighting (levels 1 to 5; Table 2), or, resists mating attempts by the male by pushing, boxing or clamping onto the male using chelae/pereopods; or (4) separate, where the female was not in contact with the male.

### Statistical analysis

Male responses to female urine (in urine blocking experiments) were analysed using Fisher exact tests. All other data were tested for normality (Kolmogorov-Smirnov test) and homogeneity (Levene test) prior to parametric analysis data. If parametric test assumptions were violated equivalent non-parametric analysis was performed. All data on the percentage urine release duration were arcsine square root transformed. Two-way ANOVA was used to investigate influence of behavioural state (dominant, subordinate, reproductive interaction) and sex on duration of urine release. *Post hoc *interaction effects were analysed using the Tukey HSD test (significance level *P *< 0.05). Paired t tests were used to compare urine output between the two interactants in each of the four treatments (Figure 1) since *P *values for these planned comparisons were not available from the two-way ANOVA. Comparisons of female urine release during different behaviours in reproductive interactions were analysed using a non-parametric Friedman ANOVA with *post hoc *Tukey tests.

To identify animals displaying high levels of aggression, an aggression index was assigned to each animal by calculating the proportion of time spent displaying aggressive behaviours at levels 4 and 5 (Table 2) in relation to all aggressive behaviours (levels 3a to 5). Pearson correlations were performed to assess if animals displaying a high aggression index also showed a high level of urine release. For correlation analysis we removed one outlier value of a male with a high aggression index that did not release any urine at all. Since all other 29 males had released at least some urine we suspect that urine visualisation was not successful in that 1 male. One-way ANOVA was used to compare the aggression index of animals between male fights, female fights and mixed-sex fights. A Tukey HSD test was used for *post hoc *comparisons.

## Authors' contributions

TB conceived the study. FCB and TB designed the study and drafted the manuscript. FCB carried out and analysed the urine visualisation experiments, TB carried out and analysed the urine-blocking experiments. Both authors read and approved the final manuscript.

## Supplementary Material

Additional file 1 M**ovie1**. This movie shows an example of male-female interaction prior to mating including female urine release. The movie shows male 'initiation', 'seizing' and the beginning of 'turning'. Female responds with aggressive behaviour (claw pushing, claw lock) accompanied by urine release and defensive behaviours (tail flip) prior to submitting to the male (receptive) at the end of the movie clip. Click here for file
